# Murine GFP-Mx1 forms nuclear condensates and associates with cytoplasmic intermediate filaments: Novel antiviral activity against VSV

**DOI:** 10.1074/jbc.RA120.015661

**Published:** 2021-01-13

**Authors:** Pravin B. Sehgal, Huijuan Yuan, Mia F. Scott, Yan Deng, Feng-Xia Liang, Andrzej Mackiewicz

**Affiliations:** 1 Departments of Cell Biology and Anatomy, New York Medical College, Valhalla, New York, USA; 2 Department of Medicine, New York Medical College, Valhalla, New York, USA; 3 Division of Advanced Research Technologies, New York University Grossman School of Medicine, New York, New York, USA; 4 Department of Medical Biotechnology, University School of Medical Sciences, Poznań, Poland; 5 Department of Diagnostics and Immunology of Cancer, Greater Poland Cancer Center, Poznań, Poland

**Keywords:** phase-separated biomolecular condensates, membraneless organelles (MLOs), interferon, myxovirus (Mx) resistance proteins, MxA, Mx1, antiviral activity, vesicular stomatitis virus, giantin-based intermediate filaments, stress granule, intermediate filament, antiviral agent, animal virus

## Abstract

Type I and III interferons induce expression of the “myxovirus resistance proteins” MxA in human cells and its ortholog Mx1 in murine cells. Human MxA forms *cytoplasmic* structures, whereas murine Mx1 forms *nuclear* bodies. Whereas both HuMxA and MuMx1 are antiviral toward influenza A virus (FLUAV) (an orthomyxovirus), only HuMxA is considered antiviral toward vesicular stomatitis virus (VSV) (a rhabdovirus). We previously reported that the cytoplasmic human GFP-MxA structures were phase-separated membraneless organelles (“biomolecular condensates”). In the present study, we investigated whether nuclear murine Mx1 structures might also represent phase-separated biomolecular condensates. The transient expression of murine GFP-Mx1 in human Huh7 hepatoma, human Mich-2H6 melanoma, and murine NIH 3T3 cells led to the appearance of Mx1 nuclear bodies. These GFP-MuMx1 nuclear bodies were rapidly disassembled by exposing cells to 1,6-hexanediol (5%, w/v), or to hypotonic buffer (40–50 mosm), consistent with properties of membraneless phase-separated condensates. Fluorescence recovery after photobleaching (FRAP) assays revealed that the GFP-MuMx1 nuclear bodies upon photobleaching showed a slow partial recovery (mobile fraction: ∼18%) suggestive of a gel-like consistency. Surprisingly, expression of GFP-MuMx1 in Huh7 cells also led to the appearance of GFP-MuMx1 in 20–30% of transfected cells in a novel cytoplasmic giantin-based intermediate filament meshwork and in cytoplasmic bodies. Remarkably, Huh7 cells with cytoplasmic murine GFP-MuMx1 filaments, but not those with only nuclear bodies, showed antiviral activity toward VSV. Thus, GFP-MuMx1 nuclear bodies comprised phase-separated condensates. Unexpectedly, GFP-MuMx1 in Huh7 cells also associated with cytoplasmic giantin-based intermediate filaments, and such cells showed antiviral activity toward VSV.

Membraneless organelles (MLOs) in the cytoplasm and nucleus formed by liquid-liquid phase-separation (LLPS) biomolecular condensates are increasingly viewed as critical in regulating diverse cellular functions (1, 2, 3, 4, 5, 6, 7, 8, 9, 10). These functions include cell differentiation, cell signaling, immune synapse function, nuclear transcription, RNA splicing and processing, mRNA storage and translation, virus replication and maturation, antiviral mechanisms, DNA sensing, synaptic transmission, protein turnover, and mitosis (reviewed in Refs. 1, 2, 3, 4, 5, 6, 7, 8, 9, 10). MLOs include the nucleolus, nucleoporin channels, nuclear speckles and paraspeckles, nuclear promyelocytic leukemia (PML) bodies, nuclear Cajal bodies, cytoplasmic processing bodies (P-bodies), germinal bodies, Balbiani bodies, Negri bodies, stress granules, translation-promoting TPA-inducible sequence (TIS) granules, and several more recent discoveries, such as condensates of synapsin, of the DNA sensor protein cyclic GMP-AMP synthase in the cytoplasm, and of active transcription-associated condensates in the nucleus (1, 2, 3, 4, 5, 6, 7, 8, 9, 10). Overall, these condensates have liquid-like internal properties (as investigated using fluorescence recovery after photobleaching (FRAP) assays) and are metastable, changing to a gel or to filaments commensurate with the cytoplasmic environment (temperature, ionic conditions, physical deformation, or cytoplasmic “crowding”), and the incorporation of additional proteins, RNA or DNA molecules, or post-translational modifications (1, 2, 3, 4, 5, 6, 7, 8, 9, 10). Indeed, specific DNA and RNA molecules participate in the assembly of many such cytoplasmic and nuclear condensates and in their functions (1, 2, 3, 4, 5, 6, 7, 8, 9, 10).

There is also an extensive literature on the changes observed in phase-separated stress granules and P-bodies in diverse virus-infected cells (11, 12, 13, 14, 15, 16). In parallel with these insights, it has now been recognized that replication and maturation of negative-strand RNA viruses (*e.g.* vesicular stomatitis (VSV), rabies (as in Negri bodies), Ebola, and measles viruses) occurs in cytoplasmic phase-separated condensates typically involving the viral nucleocapsid (N) protein with or without additional viral proteins (11, 17, 18, 19, 20, 21, 22, 23). Even the replication of a DNA virus, such as Epstein–Barr virus, involves phase-separated nuclear bodies (24). Very recently, the N protein of SARS-CoV-2 virus, a positive-strand RNA virus that replicates in the cytoplasm, has been reported to form phase-separated liquid droplets in cell-free assays by a mechanism-catalyzed viral RNA (11, 25, 26, 27, 28).

We recently reported that the human interferon (IFN)-inducible “myxovirus resistance protein A” (MxA), which displays antiviral activity against several different classes of RNA- and DNA-containing viruses (29, 30, 31), exists in the cytoplasm in membraneless metastable condensates in structures that include respective viral nucleocapsid proteins (9, 10, 32, 33), confirming a previous report from 2002 of MxA in membraneless cytoplasmic structures together with La Crosse virus N protein (34). Mx proteins are a family of large dynamin-like GTPases of molecular weight in the size range 60–70 kDa that readily oligomerize (29, 30, 31). Whereas human MxA has a cytoplasmic localization, the murine Mx1 ortholog predominantly localizes in “Mx domains” or bodies in the nucleus (29, 30, 31). Consistent with its nuclear localization, murine Mx1 has an antiviral activity against orthomyxoviruses (such as influenza A virus (FLUAV)), which have a nuclear step in their growth cycle, but is reported to be inactive against rhabdoviruses (such as vesicular stomatitis virus (VSV)), which replicate exclusively in the cytoplasm (29, 30, 31, 35, 36, 37). In contrast, human MxA shows antiviral activity toward both FLUAV and VSV (29, 30, 31).

To clarify the nomenclature of the Mx protein family, we adopt the gene lineage tracing presented by Busnadiego *et al.* (38) as follows. Most mammalian Mx proteins are formed from two distinct gene lineages (MxA or MxB) that arose from an ancient duplication event (31). Thus, humans have two Mx proteins—MxA and MxB (some investigators call these human Mx1 and human Mx2, respectively). Although mice also have two Mx proteins, Mx1 and Mx2, both of these are paralogous members of the human MxA lineage (31). The genuine ortholog of human MxB has been lost in rodent and felid lineages. Thus, human MxB (which is also called human Mx2) and murine Mx2 are *not* orthologous. In this article, we use MxA or HuMxA for the human protein and use Mx1 or MuMx1 for the orthologous murine protein.

Human MxA forms disparate membraneless structures solely in the cytoplasm and has antiviral activity toward several RNA- and DNA-containing viruses, including orthomyxo- and rhabdoviruses (29, 30, 31, 32, 33, 34, 35), whereas human MxB is mainly associated with the cytoplasmic side of nuclear pores and additional cytoplasmic membraneless structures, and the full-length MxB has antiviral activity against HIV and other lentiviruses by blocking entry of viral components into the nucleus (39, 40, 41, 42, 43) (see Ref. 44 for a recent overview). Murine Mx1 is mainly in nuclear bodies (it has a C-terminal nuclear localization signal (NLS) (45, 46, 47)), whereas murine Mx2 is mainly in cytoplasmic structures (29, 30, 31, 35, 36, 37). In terms of antiviral activity, reports in the literature indicate that the nuclear murine Mx1 has antiviral activity toward FLUAV but not VSV, whereas the cytoplasmic “granular” murine Mx2 has antiviral activity toward VSV but not FLUAV (29, 30, 31, 35, 36, 37, 48). Parenthetically, rats have three Mx proteins, Mx1, Mx2, and Mx3 (35, 36, 38, 49). Rat Mx1 and rat Mx2 are orthologs of human MxA and are nuclear and cytoplasmic, respectively (35, 36, 37, 49). However, the nuclear-predominant rat Mx1 has antiviral activity toward both FLUAV and VSV, whereas the cytoplasmic-predominant rat Mx2 is antiviral toward VSV only (35, 36, 37, 49). Rat Mx3, which is mainly dispersed in the cytoplasm, has little apparent antiviral activity (35, 36, 37, 49).

Engelhardt *et al.* (50, 51) observed that murine Mx1 nuclear bodies sometimes overlapped with or were in juxtaposition to PML bodies, nuclear speckles, and Cajal bodies. The latter three structures have all now been identified as phase-separated biomolecular condensates involved in RNA handling (1, 2, 3, 4, 5, 6, 7, 8, 9, 52). Curiously, there was no obligatory relationship between nuclear Mx1 bodies and PML bodies in that the formation of Mx1 bodies was not altered in *PML*^−/−^ cells (51).

In the present study, we investigated whether nuclear murine GFP-Mx1 bodies might also consist of phase-separated biomolecular condensates. The data obtained confirmed that murine GFP-Mx1 nuclear bodies had the properties of phase-separated biomolecular condensates in the nuclear compartment. Unexpectedly, we discovered that murine GFP-Mx1 was also associated with novel giantin-based intermediate filaments and with cytoplasmic bodies in Huh7 hepatoma cells. Remarkably, such cells expressing murine GFP-Mx1 in the cytoplasm, but not those containing only GFP-MuMx1 nuclear bodies, displayed a strong antiviral phenotype against the rhabdovirus VSV.

## Results

### Murine GFP-Mx1 forms nuclear bodies

**Figure 1:**
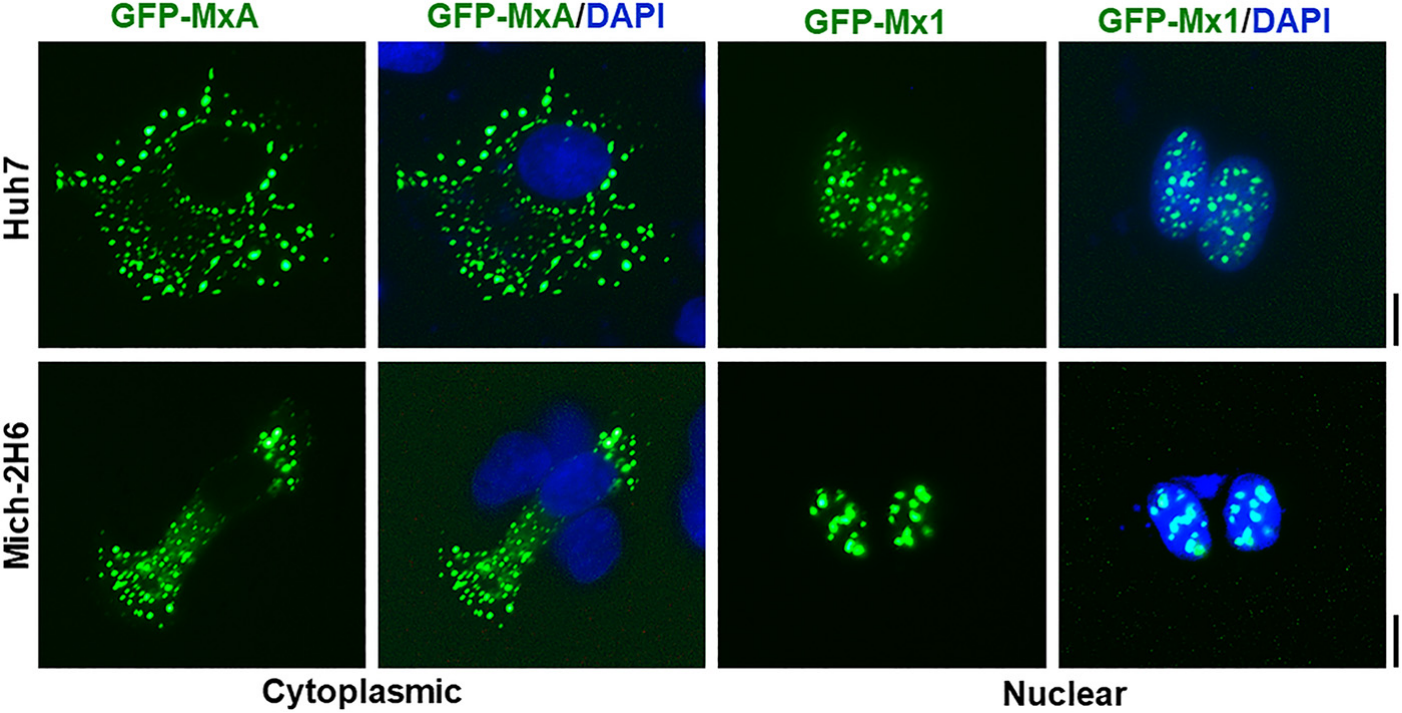
**Comparison of subcellular localization of human GFP-MxA with murine GFP-Mx1 in two different cell lines.** Cultures of Huh7 and Mich-2H6 cells in 35-mm plates were transiently transfected with expression vectors for human GFP-MxA or murine GFP-Mx1, fixed using 4% paraformaldehyde 2 days later, additionally stained with DAPI to visualize the nuclei, and imaged using two-color fluorescence. The figure illustrates representative cells. None of the cells transfected with human GFP-MxA vector showed any MxA in nuclei; 70–80% of Huh7 cells transfected with GFP-Mx1 showed only nuclear Mx1 (for cells with cytoplasmic GFP-Mx1 structures, see Figs. 7–9 and Fig. S2); almost all Mich-2H6 cells transfected with GFP-Mx1 showed only nuclear GFP-Mx1 bodies. *Scale bar*, 10 μm.

**Figure 2:**
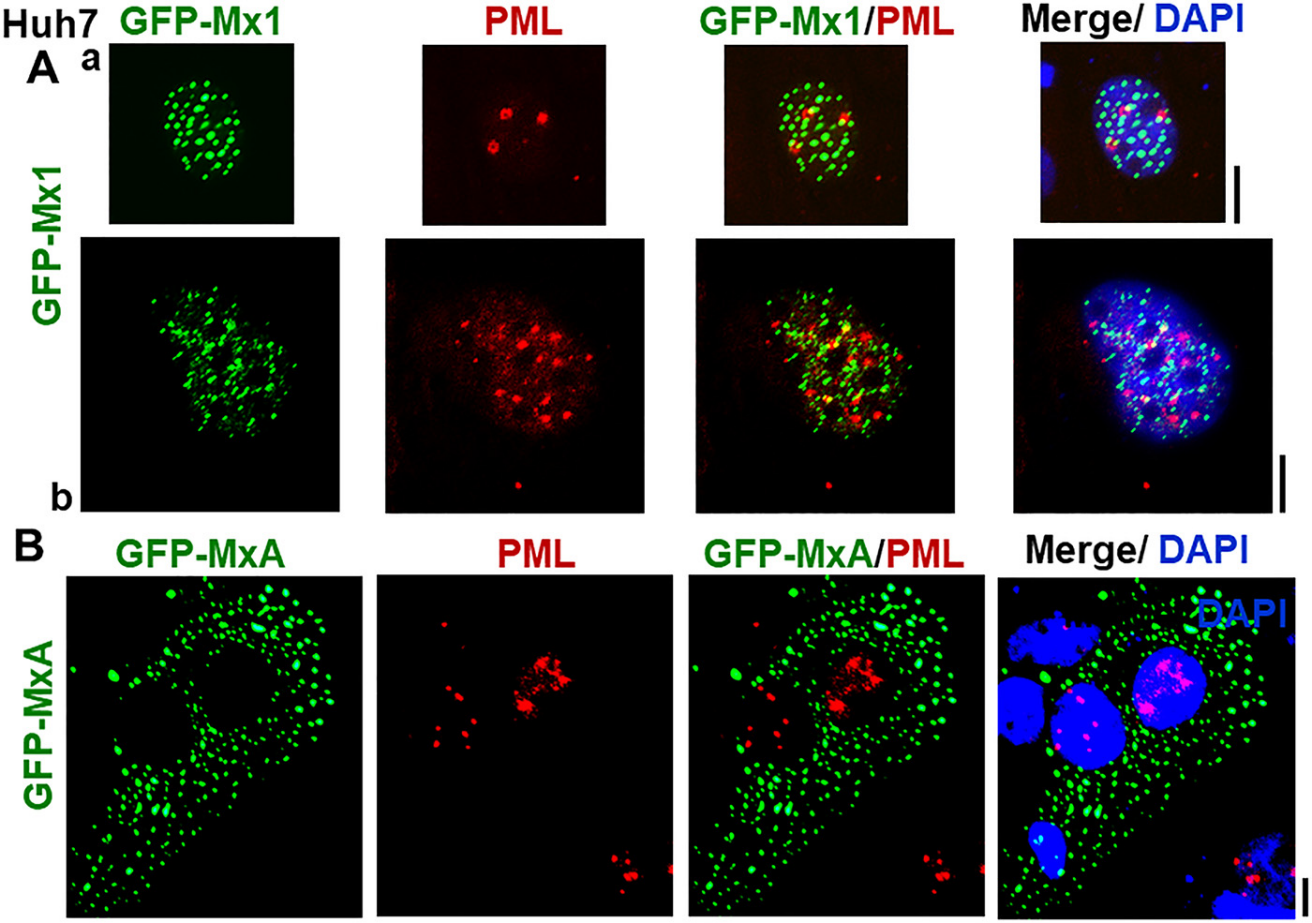
**Lack of colocalization of nuclear murine GFP-Mx1 with nuclear PML bodies.** Cultures of Huh7 cells in 35-mm plates were transiently transfected with expression vectors for murine GFP-Mx1 (*A*) or human GFP-MxA (*B*), fixed using 4% paraformaldehyde 2 days later, probed for PML using immunofluorescence methods, additionally stained with DAPI to visualize the nuclei, and imaged using three-color fluorescence. The figure illustrates representative cells. Pearson's correlation coefficients *R* (after Costes' automatic thresholding) in *Aa*, *Ab*, and *B* were 0.0, 0.25, and 0.0, respectively. *Scale bars*, = 10 μm.

In contrast to the transient expression of human GFP-MxA in Huh7 hepatoma and Mich-2H6 melanoma cells which led to formation of cytoplasmic condensates of MxA (Fig. 1, *left half*), the transient expression of murine GFP-Mx1, as expected (29, 30, 31), led to formation of nuclear bodies in most cells (Fig. 1, *right half*). These data confirm the localization of these respective proteins as reported in the literature (29, 30, 31). Moreover, in confirmation of the observations of Engelhardt *et al.* (51), the nuclear bodies formed by murine GFP-Mx1 in Huh7 cells were distinct from nuclear PML bodies (Fig. 2*A*). As a negative control, cytoplasmic GFP-MxA condensates also did not include PML (Fig. 2*B*).

### Disassembly of murine GFP-Mx1 nuclear bodies by 1,6-hexanediol

**Figure 3:**
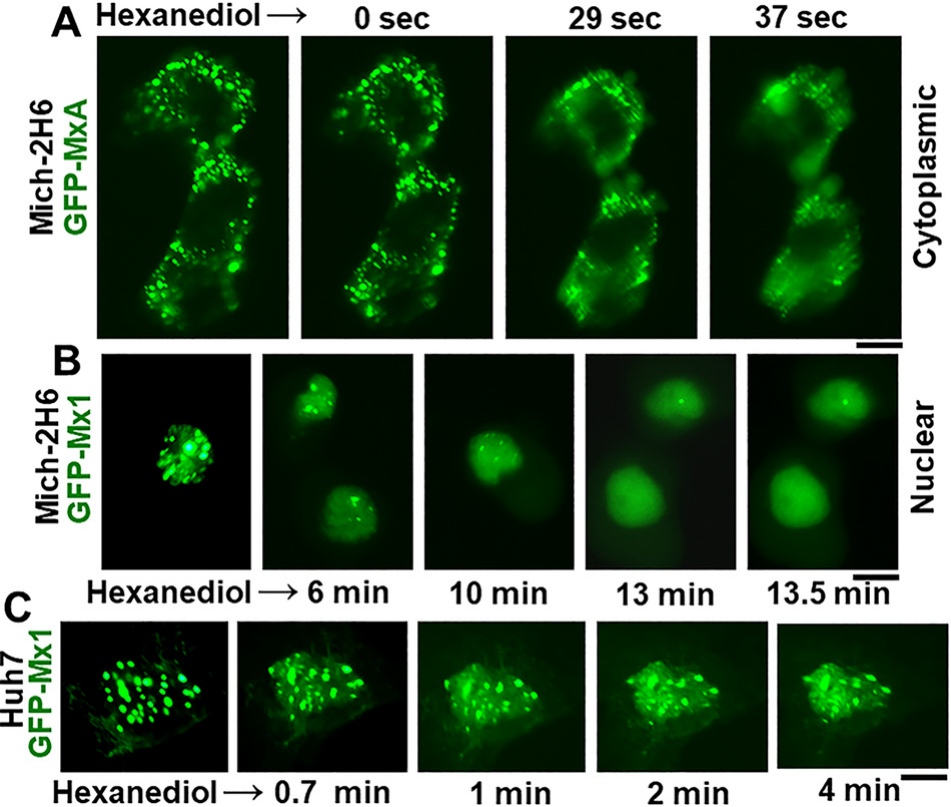
**Disassembly of nuclear GFP-Mx1 bodies by 1,6-hexanediol.** Huh7 and Mich-2H6 cells were transfected with either GFP-MxA vector (as a positive control) or GFP-Mx1 vector. 1–2 days later, the live cells were first imaged in PBS, and then the culture medium was changed to PBS supplemented with 5% (w/v) 1,6-hexanediol followed by imaging at different times thereafter (9, 33). *A*, positive control verifying the rapid disassembly of cytoplasmic GFP-MxA condensates (9, 33) in Mich-2H6 cells. *B* and *C*, a somewhat slower disassembly of GFP-Mx1 nuclear bodies after hexanediol exposure in both Mich-2H6 and Huh7 cells. *Scale bar*, 10 μm.

Rapid disassembly within 1–5 min of exposing cells to 1,6-hexanediol (3–5%, w/v) in isotonic buffer is often used as a test of the liquid-like property of structures (6, 9, 33, 53, 54, 55). Hexanediol disrupts weak hydrophobic interactions that are required to preserve the structure of phase-separated condensates (53, 54, 55). The data in Fig. 3*A* represent a positive control for the rapid disassembly effect of hexanediol on cytoplasmic condensates of GFP-MxA in Mich-2H6 cells. Fig. 3 (*B* and *C*) shows illustrative images of the disassembly of GFP-Mx1 nuclear structures when Mich-2H6 and Huh7 cells were exposed to hexanediol. Compared with the rapid disassembly of cytoplasmic human GFP-MxA condensates (in less than 1 min; Fig. 3*A*), the hexanediol-induced disassembly of nuclear murine GFP-Mx1 is relatively slower (in 4–10 min; Fig. 3, *A* and *B*). Overall, these hexanediol-triggered disassembly data provide evidence for GFP-Mx1 nuclear bodies to be LLPS condensates (9, 33, 53, 54).

### Disassembly of GFP-Mx1 nuclear bodies by hypotonicity and reassembly by isotonicity

We and others reported previously that membraneless phase-separated condensates of GFP-MxA in the cytoplasm, IL-6–induced cytoplasmic and nuclear GFP-STAT3 condensates, or FLUAV maturation structures in the cytoplasm were rapidly disassembled (within 1–3 min) in cells swollen following exposure to hypotonic buffer (9, 23, 33, 56). We further showed that a return of such cells to isotonic buffer caused rapid reassembly of MxA but in new structures different from the original ones (9, 33, 56). We have used the hypotonic disassembly–isotonic reassembly test to investigate whether GFP-Mx1 nuclear bodies might also represent such condensates.

**Figure 4:**
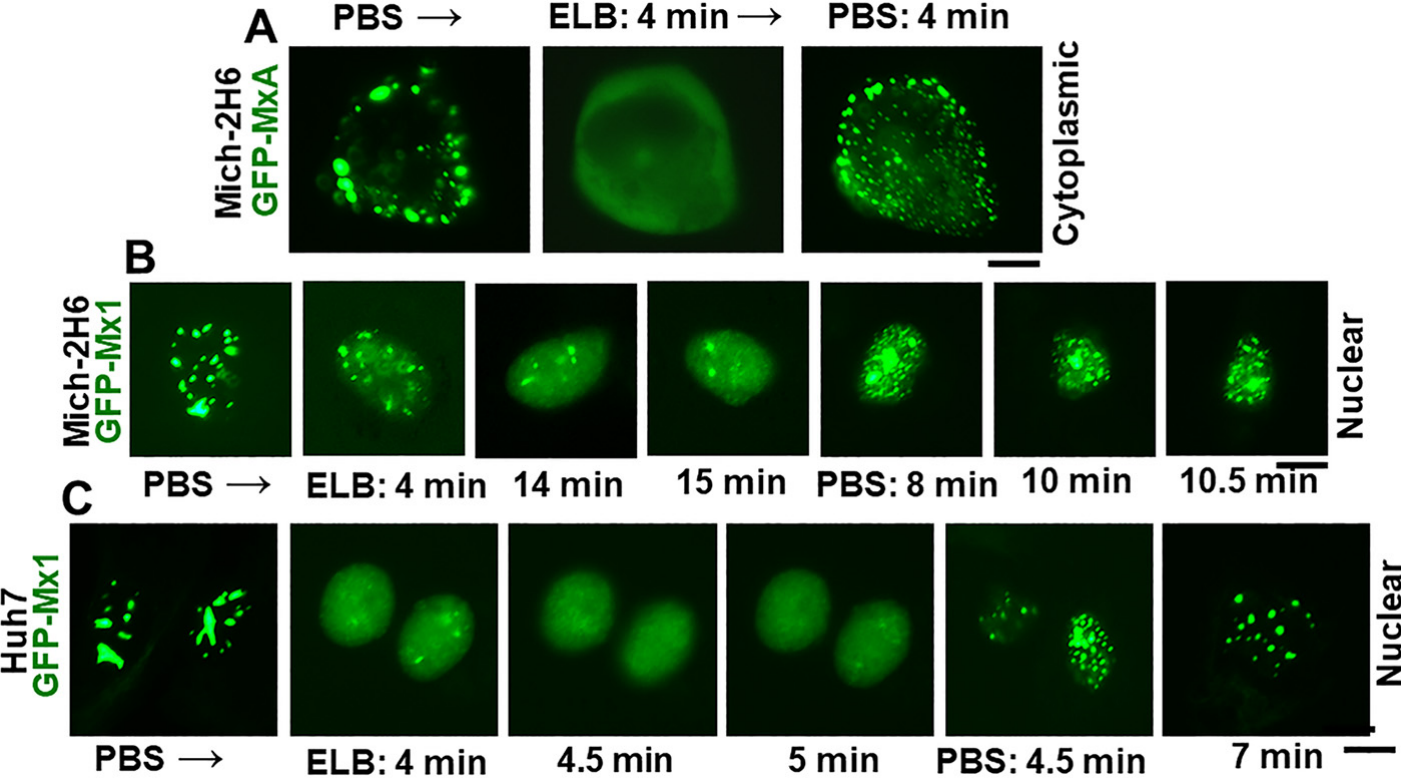
**Hypotonic disassembly and isotonic reassembly of nuclear GFP-Mx1 bodies.** Mich-2H6 or Huh7 cells were transiently transfected either with the GFP-MxA vector (as a positive control) or the GFP-Mx1 vector. 1–2 days later, the live cells were first imaged in PBS, and then the culture medium was changed to hypotonic ELB (40 mosm) followed by imaging at different times thereafter (9, 33). After 10–15 min, the culture medium was changed to isotonic PBS, and live-cell imaging continued for another 10–15 min. The figure illustrates representative cells at different time points. *A*, positive control verifying the rapid disassembly of cytoplasmic GFP-MxA condensates (9, 33) in Mich-2H6 cells upon hypotonic exposure and reassembly of the condensates upon shifting the culture medium to isotonic PBS. A time-lapse movie of this experiment is shown in Movie S1. *B* and *C*, a somewhat slower disassembly of GFP-Mx1 nuclear bodies after hypotonic exposure and isotonic reversal in both Mich-2H6 and Huh7 cells. *Scale bar*, 10 μm.

As a negative control, Fig. S1 shows that hypotonic-isotonic cycling had no effect on the dispersed N1-GFP protein expressed by itself in Huh7 cells. As another negative control, Fig. 5*A* in Ref. 56 showed that dispersed cytoplasmic GFP-tagged STAT3 expressed in Huh7 cells also showed no change when cycled through hypotonic and isotonic buffers (in the absence of the addition of IL-6). In contrast, as a positive control, the data in Fig. 4*A* and Movie S1 show the rapid disassembly of cytoplasmic GFP-MxA condensates in Mich-2H6 cells. Moreover, Fig. 4*A* and Movie S1 also show the rapid reassembly of GFP-MxA in new condensates upon returning the cultures to isotonicity. Fig. 4 (*B* and *C*) show illustrative images revealing the disassembly and reassembly of GFP-MuMx1 nuclear bodies following exposure of respective cells to hypotonic buffer and then return to isotonic buffer in both Mich2-H6 and Huh7 cells. These data further support the inference that GFP-MuMx1 nuclear bodies represent phase-separated condensates.

**Figure 5:**
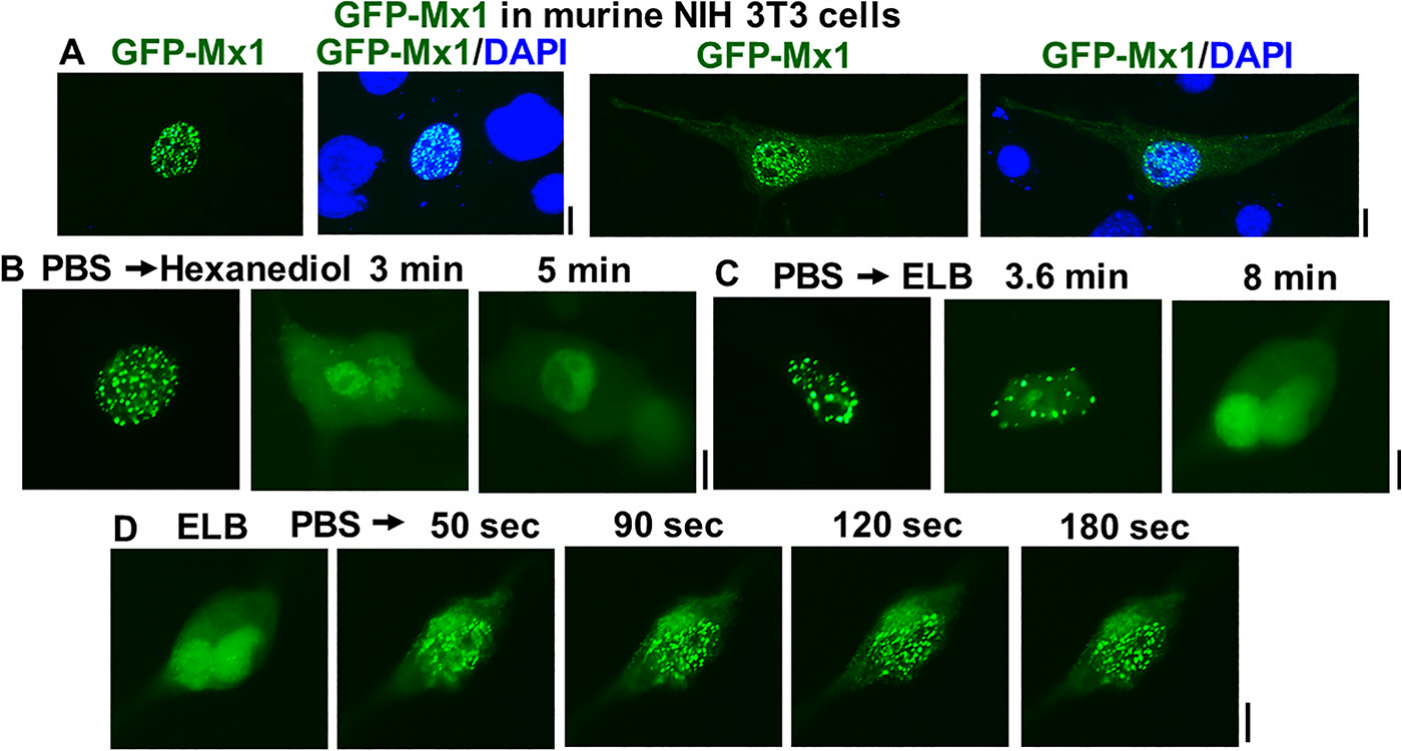
**Disassembly of murine GFP-Mx1 nuclear bodies in murine NIH 3T3 cells by 1,6-hexanediol and hypotonicity.** NIH 3T3 cells in replicate 35-mm cultures were transiently transfected with the GFP-Mx1 expression vector. One day later, one culture was fixed with 4% paraformaldehyde, stained with DAPI, and imaged (*A*). Live-cell imaging was first carried out using the other cultures first in isotonic PBS, followed by the addition of 1,6-hexanediol in PBS (*B*), or switching to hypotonic ELB (*C*). *B* and *C*, representative live-cell images at the indicated times. 10 min after adding hypotonic ELB to the culture used in *C*, it was switched to isotonic PBS, and the same cell was imaged for the next 3 min (*D*). *Scale bars*, 10 μm.

### Nuclear bodies of murine GFP-Mx1 in murine NIH 3T3 cells

Fig. 5 summarizes the phase-separation properties of nuclear bodies formed by expressing murine GFP-Mx1 in cells of the homologous species (murine NIH 3T3 cells). More than 90% of the transiently transfected GFP-Mx1–expressing 3T3 cells showed GFP-Mx1 in nuclear bodies (Fig. 5*A*). These nuclear bodies were disassembled by 1,6-hexanediol (Fig. 5*B*) and by exposing cells to hypotonic buffer (Fig. 5*C*). Moreover, after hypotonic disassembly, the GFP-Mx1 bodies reassembled rapidly (within 2–3 min) upon exposing the cells to isotonic PBS. Thus, overall, the phase-separation properties of murine GFP-Mx1 nuclear bodies were comparable in both human and murine cells (Figure 3, Figure 4, Figure 5).

### FRAP assays on murine GFP-Mx1 nuclear bodies

**Figure 6:**
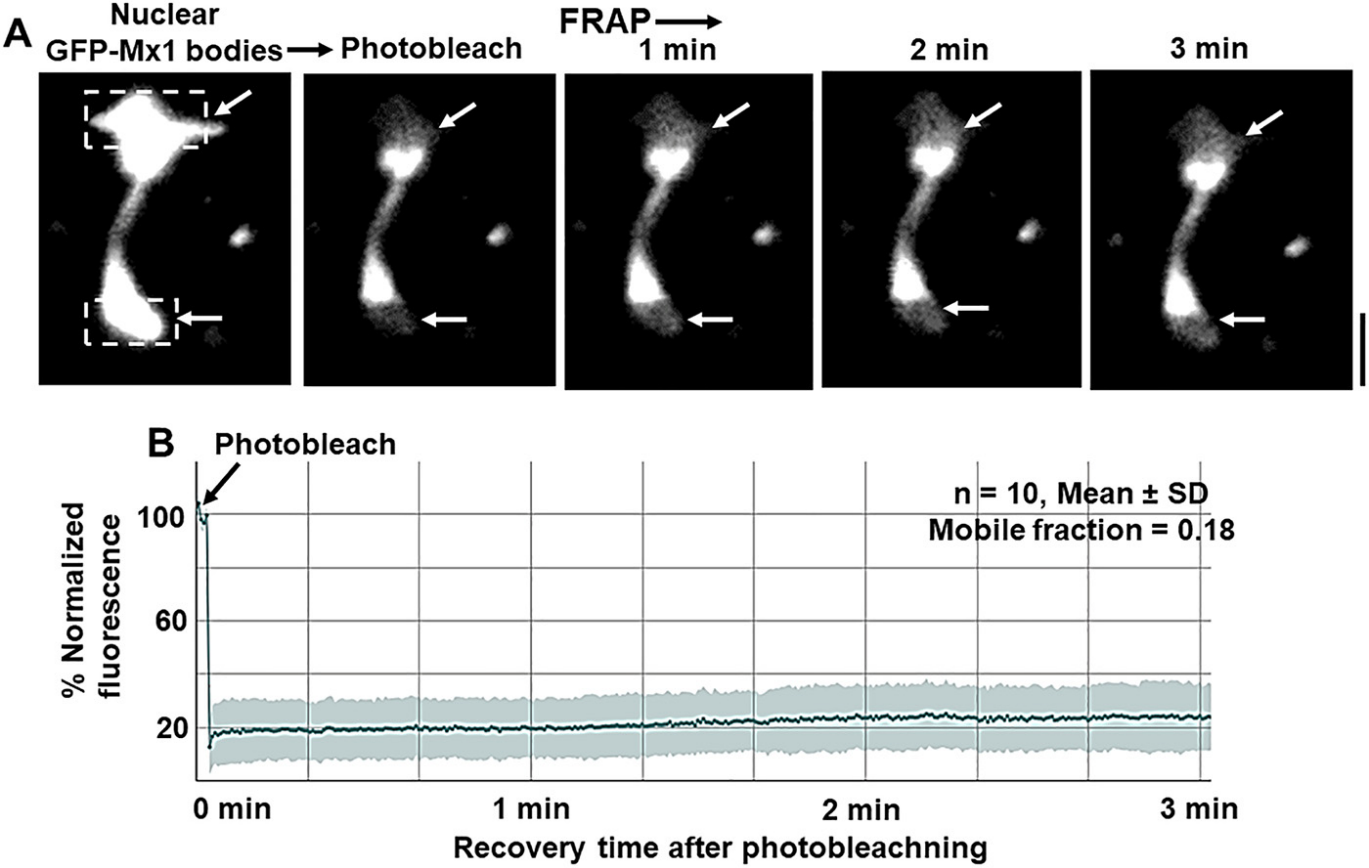
**FRAP analyses of GFP-Mx1 in nuclear bodies.** Huh7 cells grown in 35-mm coverslip-bottom plates (MatTek) were transiently transfected with the GFP-Mx1 vector, and cells were imaged 2 days later using the Zeiss 880 confocal microscopy system. Nuclear bodies in selected cells were subjected to photobleaching (*dashed rectangles*), and the recovery from bleaching was monitored for 3 min (*A*); *scale bar*, 5 μm. Movie S2 is the time-lapse movie corresponding to the images in *A*. *B* summarizes numerical data derived from 10 such experiments.

Live-cell assays quantitating the speed of fluorescence recovery after photobleaching (FRAP) provide insight about the internal miscibility of GFP-tagged proteins within a condensate (1, 2, 3, 4, 5, 7, 9); rapid recovery indicates a more liquid interior, and slower recovery suggests a more gel-like condensate (1, 2, 3, 4, 5, 7, 9). Thus, FRAP assays were carried out to investigate the mobility of GFP-Mx1 within the nuclear bodies. Fig. 6*A* and Movie S2 illustrate one example of this assay, showing the photobleaching of the top and bottom parts of an elongated GFP-MuMx1 double nuclear structure followed by live-cell monitoring of recovery over the next 3 min. Fig. 6*B* compiles together several such assays (*n* =10). Taken together, the data show a slow recovery after photobleaching (mobile fraction = 18%), consistent with a gel-like consistency of the condensate (7, 9). This observation is reminiscent of the low mobility of GFP-HuMxA within cytoplasmic condensates (mobile fraction ∼24%) (33). Thus, both GFP-HuMxA cytoplasmic condensates and GFP-MuMx1 nuclear condensates appear to have a gel-like internal consistency. As a positive control, we have confirmed >70% mobile fraction in a FRAP assay when photobleaching soluble cytoplasmic GFP-STAT3 (33).

### Murine GFP-Mx1 is associated with a novel cytoplasmic giantin-based intermediate filament meshwork

**Figure 7:**
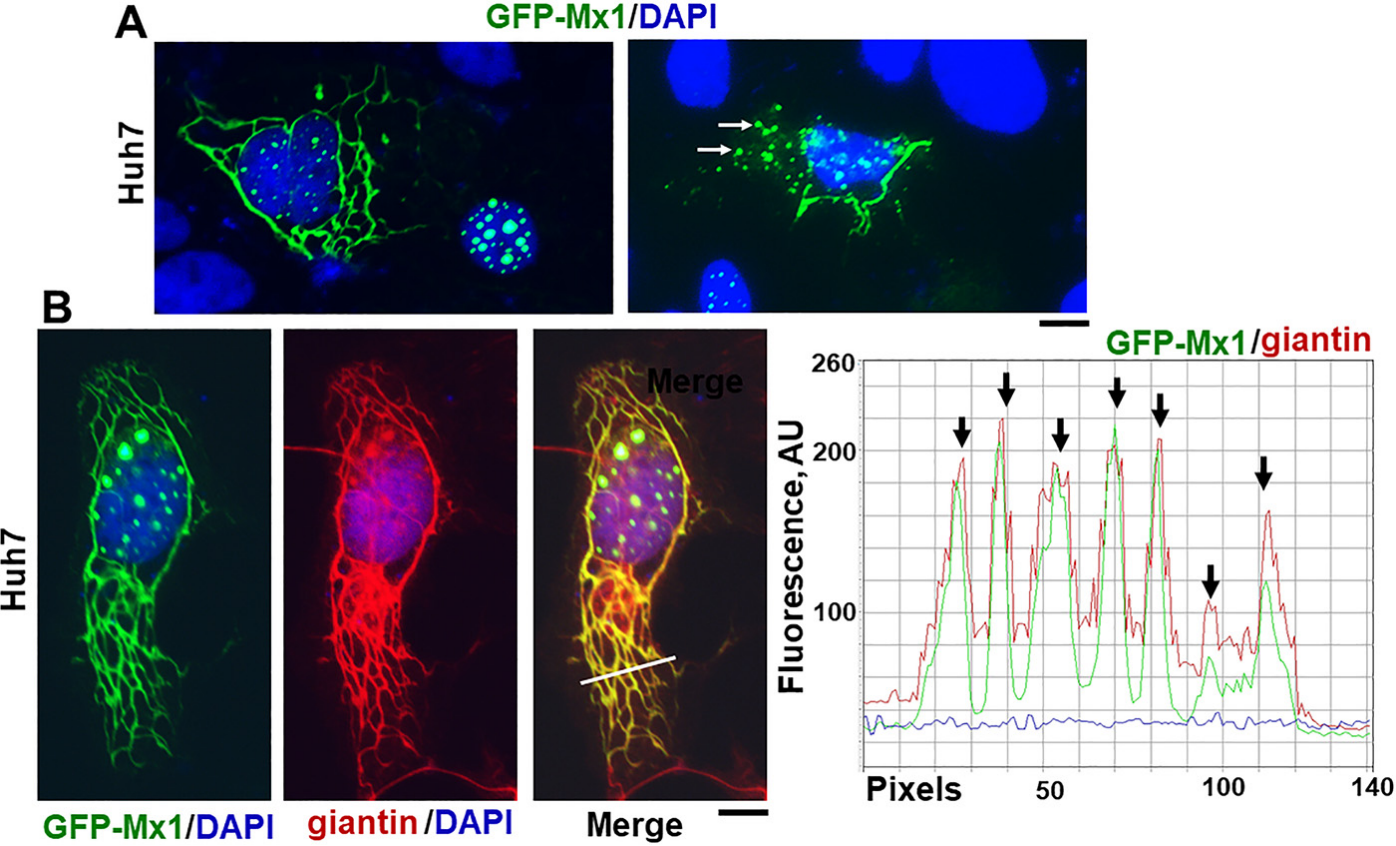
**Murine GFP-Mx1 localized to cytoplasmic structures in human Huh7 cells.** Cultures of Huh7 cells in 35-mm plates were transiently transfected with the expression vector for murine GFP-Mx1. 2 days later, the cultures were fixed using 4% paraformaldehyde, permeabilized using the 0.05% Triton buffer, and stained with DAPI to visualize the nuclei and imaged using two-color fluorescence. In *A*, the *left image* shows two adjacent cells—one showing GFP-Mx1 exclusively in nuclear bodies and one cell with GFP-Mx1 present extensively in cytoplasmic filaments and cytoplasmic structures. Similarly, the *right image* in *A* shows adjacent cells—one with extensive cytoplasmic filaments and cytoplasmic bodies of GFP-Mx1—and part of a nucleus with only nuclear GFP-Mx1 bodies (*bottom left*). Approximately 20–30% of transfected cells showed cytoplasmic murine GFP-Mx1 (also see Fig. S2). *B* summarizes a three-color immunofluorescence analysis of the cytoplasmic GFP-Mx1 filamentous structures for intermediate filaments (using the anti-giantin pAb). *Red* and *green* pixel fluorescence was measured along the *white line* in the merged image in *B*, and the data are summarized in the plot to the *right* of the image. *Arrows* in the plot indicate colocalization of red and green filaments. *Scale bars*, 10 μm.

Previous investigators have emphasized that murine Mx1 was exclusively observed in the nucleus (29, 30, 31, 35, 36, 37, 50, 51). In the present experiments, we unexpectedly discovered that murine GFP-Mx1 expressed in Huh7 cells formed extensive cytoplasmic filamentous meshwork and often were in cytoplasmic bodies in 20–30% of the transfected cells. Fig. 7*A* and Fig. S2 (A–C) show several examples of cells with nuclear bodies only (60–70% of transfected cells), cells with cytoplasmic filaments only (20–30% of cells), and cells with both (10–15%). The cytoplasmic appearance of GFP-MuMx1 was not a result of “overexpression” in that often cells with only the cytoplasmic GFP-MuMx1 meshwork displayed similar or even lesser fluorescence intensity even in the same image compared with cells with nuclear bodies (Fig. S2, images in *A–C* and quantitation in *D*). As controls, we have verified that Huh7 cells expressing only the GFP protein or expressing GFP-STAT3 (a soluble protein) do not show any cytoplasmic or nuclear structures (Fig. S1 and Fig. 5*A* in Ref. 56).

The GFP-MuMx1 filaments in Huh7 cells were identified to be novel giantin-based intermediate filaments using giantin and vimentin as markers for this cytoskeleton network. We have previously reported that the elongated protein giantin was observed in Huh7 cells not only in the Golgi elements but also in intermediate filaments (33). As further verification, two different anti-giantin pAbs revealed that this meshwork existed in Huh7 cells but not in COS7 cells (Fig. S3), that a relevant giantin peptide but not an irrelevant STAT2 peptide competed off this immunofluorescence (Fig. S4), and that both giantin and vimentin colocalized in these filaments (Fig. S5). As an additional feature, in cultures of Huh7 cells, the giantin-containing intermediate filaments extended between adjacent cells, perhaps along intercellular nanotubes (Fig. S6). We emphasize that none of the Huh7 cells illustrated in Figs. S3–S6 had been transfected with any Mx1 or MxA expression vector; the giantin-based intermediate filaments *per se* preexisted in Huh7 cells prior to transfection with the GFP-Mx1 expression vector.

**Figure 8:**
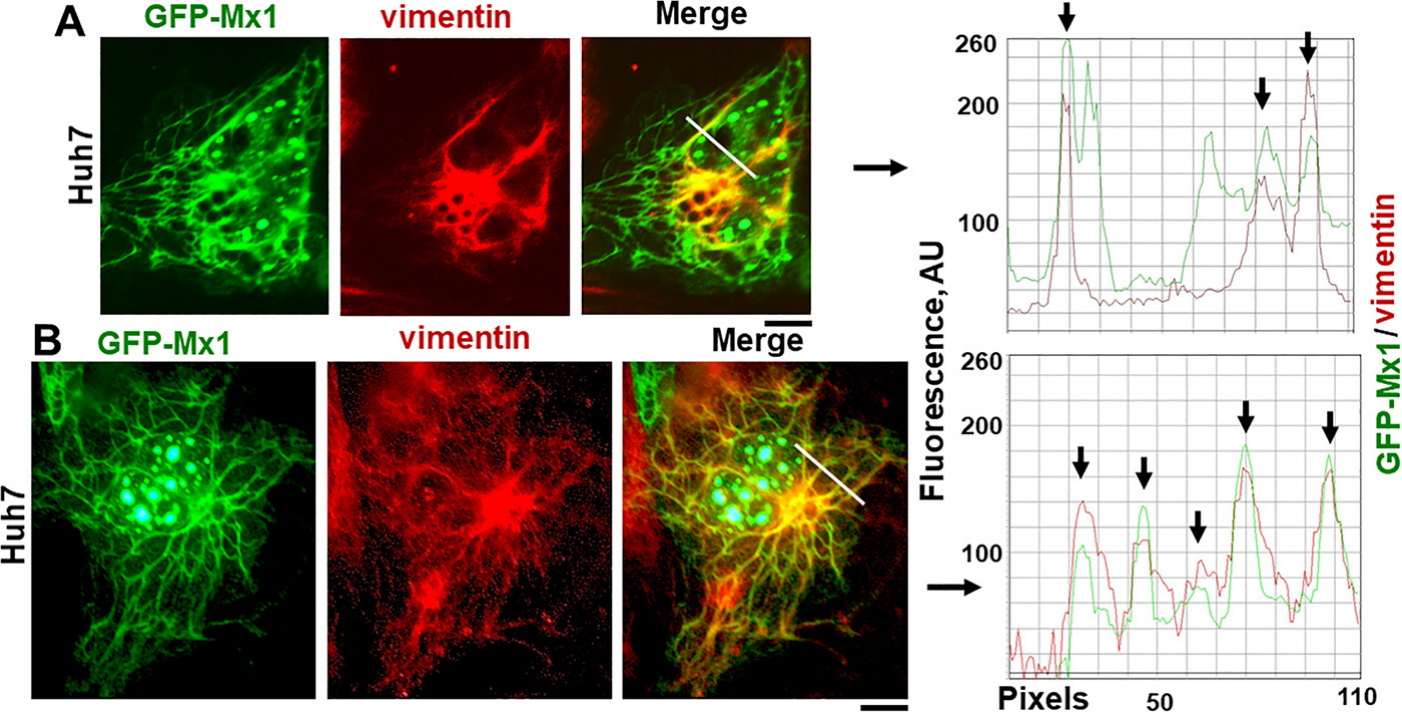
**Murine GFP-Mx1 localized to intermediate filaments in human Huh7 cells.** Cultures of Huh7 cells in 35-mm plates were transiently transfected with the expression vector for murine GFP-Mx1. 2 days later, the cultures were fixed using 4% paraformaldehyde, permeabilized using the 0.05% Triton buffer, and immunostained with anti-vimentin mAb to visualize intermediate filaments and then with DAPI to visualize the nuclei. The cultures were imaged using three-color fluorescence. *A* and *B*, two examples of cells with GFP-Mx1 in cytoplasmic filamentous structures, some of which also overlap vimentin filaments. Fluorescence in *red* and *green* pixels along the *white lines* in the merged images in *A* and *B* are plotted to the *right* of the respective images. *Arrows*, colocalized filamentous structures (GFP and vimentin). *Scale bars*, 10 μm.

Fig. 7*B* shows colocalization of GFP-MuMx1 with the giantin-based intermediate filaments. The line-scan data show clearly that GFP-Mx1 filaments were also giantin-positive. In Fig. 8 (*A* and *B*), we evaluated the colocalization of vimentin with GFP-MuMx1 filaments. It is apparent that portions of the GFP-MuMx1 cytoplasmic meshwork were vimentin-positive. Taken together, the data in Figs. 7*B* and 8 (*A* and *B*) (together with the supporting data in Figs. S3–S6) identify the cytoplasmic GFP-MuMx1 filamentous meshwork in Huh7 cells as corresponding to a novel giantin-based intermediate filament cytoskeleton. Interestingly, we have previously reported that human GFP-MxA condensates also associated with this giantin-based intermediate filament meshwork in the cytoplasm of Huh7 cells (9, 33).

**Figure 9:**
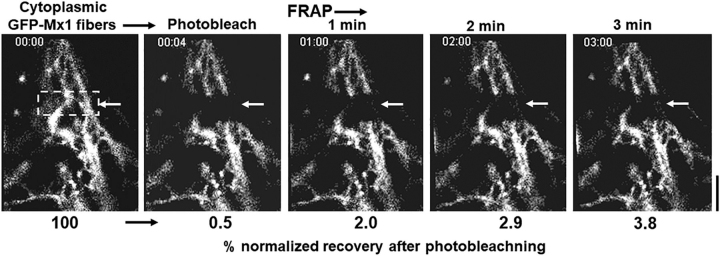
**FRAP analyses of GFP-Mx1 in cytoplasmic filamentous meshwork.** Huh7 cells grown in 35-mm coverslip-bottom plates (MatTek) were transiently transfected with the GFP-Mx1 vector, and cells were imaged 2 days later using the Zeiss 880 confocal microscopy system. A rectangular section (*dashed lines*) of a cell with GFP-Mx1 in cytoplasmic filaments was subjected to photobleaching, and the recovery from bleaching was monitored for 3 min; *scale bar*, 20 μm. The *numerals below* each *panel* summarize the percentage normalized recovery after photobleaching. Movie S3 is the time-lapse movie corresponding to this experiment.

FRAP assays confirmed that photobleaching of GFP-Mx1 in these cytoplasmic filaments led to long-lived bleaching with little detectable recovery consistent with these filaments representing a long-lived cytoskeletal structure (Fig. 9 and Movie S3). Moreover, murine GFP-Mx1 associated with cytoplasmic filaments was not disassembled by either 1,6-hexanediol or hypotonicity (data not shown).

### Antiviral activity of murine GFP-Mx1 against VSV

**Figure 10:**
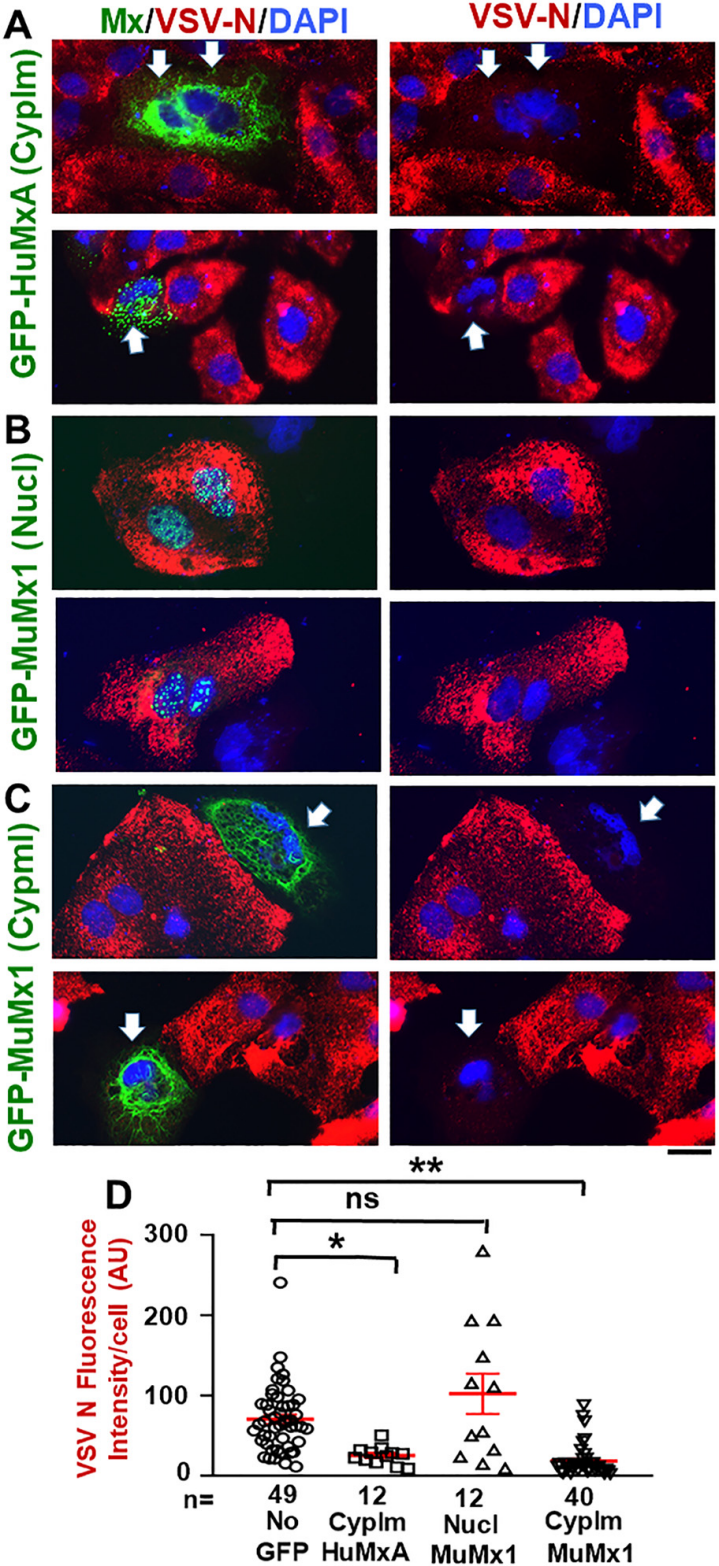
**Antiviral phenotype of Huh7 cells with nuclear or cytoplasmic GFP-MuMx1 toward VSV.** Huh7 cells (∼2 × 10^5^ cells/35-mm plate), transfected with the GFP-HuMxA or GFP-MuMx1 expression vectors 2 days earlier, were replenished with 0.25 ml of serum-free Eagle's medium and then 20 µl of a concentrated VSV stock of the WT Orsay strain added (corresponding to multiplicity of infection >10 pfu/cell). The plates were rocked every 15 min for 1 h followed by the addition of 1 ml of full culture medium. The cultures were fixed with 4% paraformaldehyde at 4 h after the start of the VSV infection, and the extent of VSV N protein expression in individual cells was evaluated using immunofluorescence methods (using the mouse anti-N mAb) and Image J for quantitation (33). *A–C*, representative cells showing the absence of any GFP or the appearance of cytoplasmic GFP-HuMxA, nuclear GFP-MuMx1, or cytoplasmic GFP-MuMx1 and the corresponding level of expression of viral N protein (*thick arrows* point to cells displaying an antiviral effect). All *scale bars*, 20 μm. *D* enumerates N protein expression in various classes of cells shown in *A–C* imaged at identical exposure settings and expressed in AU/cell. *n*, number of cells evaluated per group in this experiment (for this evaluation, cells with only cytoplasmic Mx1 were combined with cells with both cytoplasmic and nuclear Mx1); *horizontal red lines* within each group indicate mean ± S.E. (*error bars*). Statistical significance was evaluated using ANOVA (Kruskal–Wallis with Dunn's post-test for multiple comparisons); *, *p* < 0.01; **, *p* < 0.001; *ns*, not significant (*p* > 0.05).

It has been previously reported that murine Mx1 did *not* have an antiviral activity toward VSV, a rhabdovirus which replicates exclusively in the cytoplasm (31, 35, 36). With the realization that a significant subset of Huh7 cells expressed GFP-MuMx1 in cytoplasmic structures (Figure 7, Figure 8), and the ability to investigate an antiviral activity toward VSV at the single-cell level using immunofluorescence methods (33, 36), we investigated whether cells expressing cytoplasmic GFP-MuMx1 might also display an antiviral phenotype toward VSV. It needs emphasis that because transient transfection methods introduce the GFP-Mx1 vector into at best only 10–15% of cells in a culture, and the mixed phenotypes (nuclear bodies alone, cytoplasmic filaments, or both in a minority of cells (see Fig. S2), it is necessary to evaluate the antiviral phenotype at the single-cell level in groups of cells tabulated in accordance with their GFP-Mx1 phenotypes. Thus, in the present antiviral experiment (Fig. 10), we used single-cell imaging methods to quantitate VSV N protein expression in three groups: cells with no GFP expression, those with GFP-Mx1 nuclear bodies, or those showing cytoplasmic filaments (with or without nuclear bodies). Huh7 cultures were first transiently transfected with the GFP-MuMx1 expression vector or a GFP-HuMxA vector as a positive control, and the cultures were challenged 2 days later with VSV at a multiplicity of infection >10 pfu/cell. The cultures were fixed 4 h later, and the cells were probed for expression of viral N protein by immunofluorescence. Images were quantitated at the single-cell level using ImageJ for the levels of expression of N protein in cells showing no transfection, cytoplasmic GFP-HuMxA, only nuclear GFP-MuMx1, or cytoplasmic GFP-MuMx1 (Fig. 10). Fig. 10 (*A–C*) shows representative examples of respective cells, whereas Fig. 10*D* summarizes the relevant quantitation. Fig. 10 (*A* and *D*) confirms the antiviral activity of HuMxA toward VSV. Fig. 10 (*B* and *D*) confirms the previous observations of Haller and colleagues (31, 35, 36) that cells expressing MuMx1 exclusively in nuclear bodies show no antiviral effect toward VSV. Remarkably, the data in Fig. 10 (*C* and *D*) reveal that Huh7 cells with cytoplasmic MuMx1 have a strong antiviral phenotype toward VSV.

## Discussion

The IFN-induced antiviral proteins human MxA and its orthologous murine Mx1 localize predominantly to different cellular compartments—the human protein is cytoplasmic, whereas the murine protein is, until now, thought to be exclusively nuclear (29, 30, 31, 35). We previously showed that human MxA formed membraneless phase-separated condensates and filaments in the cytoplasm (9, 32, 33). In the present study, we provide evidence that the nuclear bodies formed by murine Mx1 are also phase-separated condensates. Moreover, FRAP analyses showed that, as with GFP-HuMxA condensates (mobile fraction: ∼24% (33)), GFP-MuMx1 nuclear condensates had a gel-like internal consistency (mobile fraction: ∼18%). Additionally, we discovered that murine GFP-MuMx1 also participated in the formation of cytoplasmic bodies and a meshwork of cytoplasmic intermediate filaments in a subset of Huh7 cells. The latter observations suggested novel functions of MuMx1 in the cytoplasm, Indeed, we observed that cells with cytoplasmic MuMx1 displayed an antiviral activity toward VSV, a virus that replicates exclusively in the cytoplasm and one that had been previously reported to be unaffected by murine Mx1 (29, 30, 31). Thus, taken together with our previous data (32, 33), the present study shows (*a*) that both human MxA and murine Mx1 give rise to phase-separated biomolecular condensates, albeit in different cellular compartments, (*b*) both human MxA and murine Mx1 exhibit association with cytoplasmic intermediate filaments, at least in Huh7 cells, and (*c*) both human MxA and murine Mx1 when cytoplasmic exhibit an antiviral activity toward VSV—a rhabdovirus that replicates and matures entirely in the cytoplasm (Table 1).

**Table 1:** Properties of different human and murine Mx proteins

Protein	Cellular localization	Illustrative antiviral activity^a^	References
Human MxA	Cytoplasmic condensates with some tethered to intermediate filaments	*Versus* both FLUAV and VSV	9, 29,30, 31, 32, 33, 34,35
Human MxB (full-length)	Cytoplasmic face of nuclear pores	*Versus* HIV; not *versus* FLUAV nor VSV	31, 39,40, 41, 42, 43,44
	Cytoplasmic condensates		Our inference
Murine Mx1	Nuclear condensates	*Versus* FLUAV; not VSV	This study and Refs. 29–31, 35, 36
	Cytoplasmic intermediate filaments and condensates	*Versus* VSV	This study
Murine Mx2	Cytoplasmic structures	*Versus* VSV; not FLUAV	31, 35–37


a See Refs. 31, 35, and 44 for detailed antiviral spectrum reported in the previous literature.

The data showing the close relationship between MxB and nucleoporins on the cytoplasmic face of nuclear pores and the involvement of MxB in regulating cargo transit through the pore channel (39, 40, 41, 42, 43) allow us to now identify MxB structures at and near the nuclear pore to also represent phase-separated biomolecular condensates (Table 1) (53, 54). Previous research focused on characterizing the physical properties of various nucleoporins has already demonstrated these proteins to have the property of LLPS (53, 54). Indeed, compounds such as hexanediol were used in cell biology to inhibit permeability through the pore channels through disruption of weak hydrophobic bonds between nucleoporin proteins in the pore condensates (53, 54, 55). Parenthetically, these observations eventually led to the more general use of disassembly by hexanediol as a test for phase-separated condensates (9, 55). Thus, the association of MxB with nuclear pore structures (39, 40, 41, 42, 43) taken together with the demonstration of nuclear pore structures to be phase-separated condensates (53, 54) points to the inference that MxB also associates with membraneless phase-separated condensates (see Fig. 2 (*E* and *F*) in Ref. 39 for examples).

Murine Mx1 typically shows antiviral activity toward FLUAV and other orthomyxoviruses that require a nuclear step in their replication, but not toward rhabdoviruses, such as VSV, which replicate entirely in the cytoplasm (29, 30, 31, 35) (Table 1). In contrast, functional murine Mx2 (isolated from feral mice) was observed to display an antiviral activity toward VSV but not FLUAV (35, 37). Commensurately, whereas murine Mx1 was observed to be in nuclear bodies, murine Mx2 was observed to be mainly in granular cytoplasmic structures (36, 37), which might perhaps also represent biomolecular condensates (Table 1).

There is extensive literature over the last 3 decades on the genetic dissection of the nuclear localization signal (NLS) in murine Mx1 (45, 46, 47). The C-terminal 19 amino acids in MuMx1 form a basic domain that has been referred to as a “weak” nuclear localization signal (45, 46, 47). This NLS was considered “weak” in that although its deletion in Mx1 inhibited nuclear localization, this domain by itself was unable to confer nuclear localization on other proteins (*e.g.* on MxA) (46). In terms of antiviral activity, Mx1 with a deleted NLS or the Glu-614 point mutation within the NLS was cytoplasmic and lost anti-FLUAV activity (46). The attachment of the T antigen NLS at the N terminus of such a deletion or point mutant restored nuclear localization and anti-FLUAV antiviral activity (46). Of relevance to the present study, such cytoplasmic NLS-deleted or point mutant murine Mx1 species did not show antiviral activity toward VSV. In light of this prior literature, our discovery of experimental conditions under which WT GFP-Mx1 was cytoplasmic and also showed antiviral activity toward VSV was unexpected (Figure 7, Figure 8, Figure 9, Figure 10).

We observed that a subset of GFP-MuMx1–expressing Huh7 cells contained cytoplasmic Mx1 structures, especially in association with novel giantin-based intermediate filaments. Such cells displayed an antiviral activity toward VSV (Fig. 10 (*C* and *D*) and Table 1). Such an antiviral activity was not observed in cells exclusively expressing GFP-MuMx1 in nuclear bodies (Fig. 10, *B* and *D*). Thus, our data confirm the previous observation that cells expressing murine Mx1 only in the nucleus do not display an antiviral phenotype toward VSV (29, 30, 31) but now extend these observations to the discovery that murine GFP-Mx1–expressing Huh7 cells with cytoplasmic Mx1 expression show antiviral activity toward VSV. These data emphasize that the subcellular localization of respective Mx proteins affects the spectrum of Mx antiviral phenotypes observed. Curiously, even when human MxA and murine Mx1 were co-expressed in the same cells, the two Mx proteins remained in their distinct cytoplasmic *versus* nuclear compartments, respectively (51).

Very recently, Steiner and Pavlovic (44) have also emphasized that the subcellular localization affects the antiviral spectrum of human MxB protein (Table 1). In the new studies, it was observed that human MxB, which has an N-terminal NLS that ferries the protein to the nuclear pore, did not have an anti-FLUAV activity (44) (Table 1). However, when this was replaced with the NLS from T antigen, the Tg- MxB generated nuclear bodies and gained an anti-FLUAV phenotype in addition to its anti-HIV phenotype. Conversely, replacing the N terminus of MxB with the 43-amino acid N-terminal disordered region in MxA generated a chimeric protein that was cytoplasmic and had an anti-FLUAV antiviral activity. Thus, remarkably, both cytoplasmic and nuclear MxB engineered in different ways had acquired a novel anti-FLUAV antiviral property not evident with the WT MxB. These studies in the literature and our present observations collectively emphasize the importance of subcellular localization in contributing to the antiviral spectrum of Mx proteins.

Additional mutational studies of human MxA show that the GTPase activity is required for its antiviral activity except for that toward hepatitis B virus (29, 30, 31, 57, 58). However, data in the literature reveal that MxA mutants lacking GTase activity can still form cytoplasmic condensates (31, 58). Mutations that cause dispersal of MxA in the cytoplasm (*e.g.* the D250N mutant) lacked antiviral activity (31, 57, 58). The Arg-645 point mutant of MxA, which was in larger cytoplasmic granules, had the unusual property of inhibiting FLUAV but not VSV, although the WT MxA showed antiviral activity toward both viruses (47). A mutational analysis of rat Mx2 showed that mutants that formed “granular” cytoplasmic structures exhibited antiviral activity toward VSV, whereas those that were “diffuse” in the cytoplasm did not (49). These data suggest that condensate formation may be important for the antiviral activity of Mx proteins.

The hypotonicity-driven disassembly of Mx protein condensates in live cells (33) (present data) highlight an unusual aspect of Mx protein chemistry. The biochemical basis for this hypotonicity-driven disassembly may reflect the effect of cytoplasmic “crowding” on higher-order protein structure or rapid changes due to hypotonicity-triggered post-translational modifications (4, 6, 9, 33). The reversal to isotonicity led to the reformation of both cytoplasmic GFP-HuMxA and nuclear GFP-MuMx1 condensates, albeit different from the original pre-hypotonicity condensates. These data suggest that the GFP-Mx proteins disperse completely under hypotonic conditions and then reassemble upon reversal of cells to isotonicity to form new phase-separated condensates. It is noteworthy that virus infection often leads to the development of intracellular edema (59, 60, 61, 62). Whether Mx proteins maintain their antiviral phenotype in the face of intracellular edema is not known.

Limitations of the present study include the fact that to investigate single Mx species one at a time and without interference with endogenous Mx species, we carried out transient transfection experiments using a vector for GFP-tagged Mx1 in heterologous human cells (Huh7 and Mich2-H6) and also in cells of homologous species (NIH 3T3). These studies remain to be extended to investigations of endogenous Mx1 species induced in MuIFN-treated murine cells. We have previously shown that, as with GFP-HuMxA transiently expressed in Huh7 cells, IFN-α–induced endogenous human MxA also forms phase-separated cytoplasmic condensates (33).

To summarize, the present data taken together with those in the literature suggest that human MxA, its murine orthologs Mx1 and Mx2, and human MxB likely have a common biophysical property—the ability to undergo LLPS (Table 1). This results in the formation of different phase-separated biomolecular condensates (also called membraneless organelles, or MLOs) in different cellular compartments. Remarkably, the discovery of the antiviral activity of GFP-MuMx1 toward VSV further emphasizes the critical contribution of differences in subcellular localization in the biology of Mx proteins.

## Experimental procedures

### Cells and cell culture

Human hepatoma cell line Huh7 (63) was a gift from Dr. Charles M. Rice (The Rockefeller University). Human Mich2-H6 melanoma cells were developed by Mackiewicz *et al.* (64). The respective cell lines were grown in DMEM supplemented with 10% (v/v) fetal bovine serum in T25 flasks (32, 33, 65, 66). NIH 3T3 cells were grown in DMEM supplemented with 10% (v/v) calf serum and 2 mm glutamine. For experiments, the cells were typically plated in 35-mm dishes without or with coverslip bottoms (32, 33).

### Plasmids and transient transfection

The GFP(1–248)-tagged full-length human MxA and the GFP-tagged full-length murine Mx1 expression vectors were gifts of Dr. Jovan Pavlovic (University of Zurich) (57, 67); the GFP tag was located on the N-terminal side of the Mx coding sequence. Plasmid vectors for expression of the N1-GFP tag only and one for expression of GFP-STAT3 were used as negative controls (56, 68). Transient transfections were carried out using just subconfluent cultures in 35-mm plates using DNA in the range of 0.3–2 μg/culture and the Polyfect reagent (Qiagen, Germantown, MD) and the manufacturer's protocol (with 10 μl of Polyfect reagent/35-mm plate).

### VSV stock and virus infection

A stock of the WT Orsay strain of VSV (titer: 9 × 10^8^ pfu/ml) was a gift of Dr. Douglas S. Lyles (Department of Biochemistry, Wake Forest School of Medicine, Winston-Salem, NC). Virus infection was carried out essentially as described by Carey *et al.* (69) as summarized by Davis *et al.* using Huh7 cells (33). Briefly, Huh7 cultures (∼2 × 10^5^ cells/35-mm plate), previously transfected with the pGFP-Mx1 expression vector (1–2 days earlier), were replenished with 0.25 ml of serum-free Eagle's medium, and 10–20 μl of the concentrated VSV stock was added (corresponding to multiplicity of infection >10 pfu/cell). The plates were rocked every 15 min for 1 h, followed by the addition of 1 ml of full culture medium. For the experiment shown in Fig. 10, the cultures were fixed at 4 h after the start of the VSV infection and immunostained for VSV N protein using an mAb provided by Dr. Douglas S. Lyles (mAb 10G4). N protein immunofluorescence (in *red*) in GFP-positive (nuclear or cytoplasmic) and GFP-negative cells was quantitated on a per cell basis as summarized by Davis *et al.* (33) and expressed in arbitrary units (AU) as integrated intensity/cell.

### Live-cell fluorescence imaging

Live-cell imaging of GFP-MxA and GFP-Mx1 structures in transiently transfected cells was carried out in cells grown in 35-mm plates using the upright Zeiss AxioImager 2 equipped with a warm (37 °C) stage and a ×40 water-immersion objective and also by placing a coverslip on the sheet of live cells and imaging using the ×100 oil objective (as above) with data capture in a time-lapse or z-stack mode (using Axiovision 4.8.1 software) (27).

FRAP experiments were performed using a Zeiss LSM880 confocal microscope with Zeiss Plan-Apochromat ×63/1.4 NA oil objective on randomly picked cells grown in coverslip-bottom 35-mm culture dishes (33). Five prebleach images were acquired before 25 cycles of bleaching with full-power argon 488-nm laser on a small area (∼3 μm^2^), which reduces intensity to ∼50%. Fluorescence recovery was monitored continuously for 180 s at 2 frames/s. All image series were aligned and registered with Fiji ImageJ's SIFT plugin, and FRAP analyses of mobile fraction and *t*_½_ were performed afterward with a Jython script (https://imagej.net/Analyze_FRAP_movies_with_a_Jython_script) incorporated into ImageJ (Fiji).

### Phase transition experiments

Live GFP-Mx1– or GFP-MxA–expressing Huh7 hepatoma cells in 35-mm plates were imaged using a ×40 water-immersion objective 2–5 days after transient transfection in growth medium or serum-free DMEM or in PBS. After collecting baseline images of Mx condensates (including time-lapse sequences), the cultures were exposed to 1,6-hexanediol (5%, w/v) in PBS or to hypotonic buffer (ELB; 10 mm NaCl, 10 mm Tris, pH 7.4, 3 mm MgCl_2_), and live-cell time-lapse imaging continued (9, 10, 33). After ∼5–10 min, the cultures exposed to hypotonic ELB were replenished with isotonic PBS and imaged for another 5–10 min.

### Immunofluorescence imaging

Typically, the cultures were fixed using cold paraformaldehyde (4%) for 1 h and then permeabilized using a buffer containing digitonin or saponin (50 μg/ml) and sucrose (0.3 m) (33); cultures for immunostaining of nuclear bodies, vimentin, or VSV N protein were permeabilized using 0.05% Triton in PBS. Single-label and double-label immunofluorescence assays were carried out using antibodies as indicated, with the double-label assays performed sequentially. Fluorescence was imaged as reported previously (32, 33, 56, 65, 66) using an erect Zeiss AxioImager M2 motorized microscopy system with Zeiss W N-Achroplan ×40/0.75 NA water-immersion or Zeiss EC Plan-Neofluor ×100/1.3 NA oil objectives equipped with a high-resolution RGB HRc AxioCam camera and AxioVision 4.8.1 software in a 1388 × 1040-pixel high-speed color capture mode. Images in z-stacks were captured using Zeiss AxioImager software; these stacks were then deconvolved and rendered in three dimensions using the 64-bit version of the Zeiss AxioVision software. Deconvolution of two-dimensional images was carried out using ImageJ (Fiji) software. Colocalization analyses were carried out using ImageJ software (Fiji), and line scans were carried out using AxioVision 4.8.1 software. High-resolution immunofluorescence/fluorescence imaging of selected cultures was carried out using a Zeiss Confocal 880 Airyscan system (×100 oil/1.46 NA objective).

### Antibody reagents

Rabbit pAb to human MxA (H-285) (sc-50509) and mouse mAb vimentin (V9) (sc-6260) were purchased from Santa Cruz Biotechnology, Inc. (Dallas, TX). Rabbit pAb to giantin (1–469 fragment) (ab24586) was purchased from Abcam Inc. (Cambridge, MA); a different rabbit pAb to giantin (1–469 fragment) (Poly19243) was also purchased from BioLegend Inc. (San Diego, CA). Mouse mAb to the VSV nucleocapsid (N) designated 10G4 was a gift from Dr. Douglas S. Lyles (Wake Forest School of Medicine, Winston-Salem, NC). Blocking peptides corresponding to human STAT2 and human giantin(1–469) were purchased from Santa Cruz Biotechnology, Inc. or custom-synthesized by GeneScript USA Inc. (Piscataway, NJ), respectively. Respective Alexa Fluor 488– and Alexa Fluor 594–tagged secondary donkey antibodies to rabbit (A-11008 and A-11012) or mouse (A-21202 and A-21203) IgG were from Invitrogen Molecular Probes (Eugene, OR).

### Statistical testing

Statistical testing was carried out (as in Fig. 10*D* and Fig. S2) using nonparametric one-way ANOVA (Kruskal–Wallis) with Dunn's post hoc test for multiple comparisons.

## Data availability

All data are contained within the article.
