# Supplementary material for: Murine GFP-Mx1 forms nuclear condensates and associates with cytoplasmic intermediate filaments: Novel antiviral activity against VSV
Source: J Biol Chem. 2021 Jan 13;295(52):18023–35. doi: 10.1074/jbc.RA120.015661 (PMC7939456; doi:10.1074/jbc.RA120.015661)
Supplement: Supplementary file 1 [file mmc1.zip › 162950_1_supp_603845_qhdjnn.pdf]

Supporting Information

Murine GFP-Mx1 forms nuclear condensates and associates with cytoplasmic intermediate filaments: novel antiviral activity against VSV

Pravin B. Sehgal[1,2,*], Hujuan Yuan[1,#], Mia F. Scott[1], Yan Deng[3], Feng-Xia Liang[3], Andrzej Mackiewicz[4]

Supporting Movies

Movie S1. Time-lapse movie of the disassembly and reassembly of cytoplasmic human GFP-MxA condensates in Mich2-H6 cells. This movie corresponds to the experiment shown in Fig. 3, Panel A.

Movie S2. Time-lapse movie of the FRAP experiment on nuclear GFP-Mx1 condensates shown in Fig. 6A.

Movie S3. Time-lapse movie of the FRAP experiment on cytoplasmic filamentous GFP-Mx1 shown in Fig. 9.

Supporting Figures

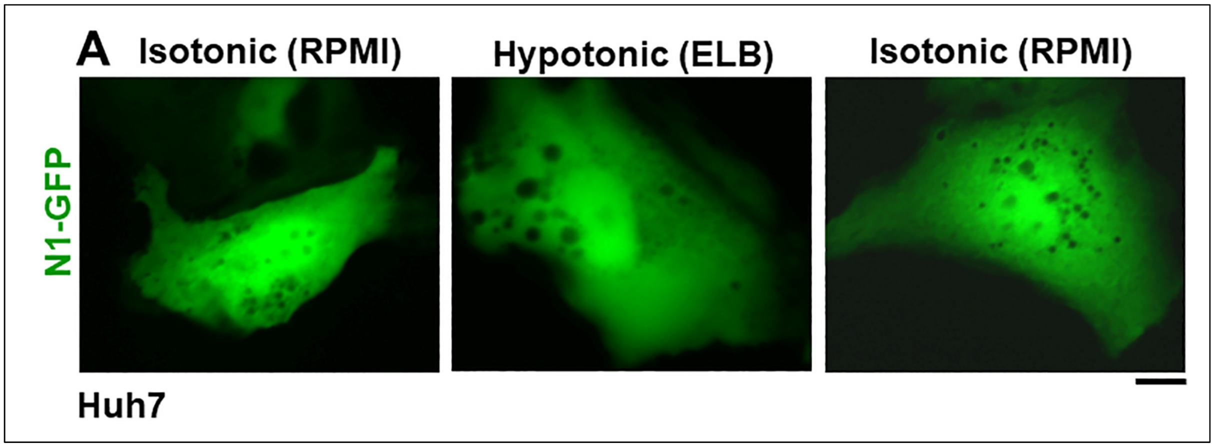

Fig. S1. N1-GFP tag expressed in huh7 cells is not affected by hypotonic-isotonic cycling. Huh7 cells were transiently transfected with the N1-GFP vector as a negative control. One day later the live cells were first imaged in full medium (RPMI), and then the culture medium was changed to hypotonic ELB buffer (40 mOsm) followed by imaging at different times thereafter (9, 33). After 10-15 min, the culture medium was changed to isotonic RPMI live cell imaging continued for another 10-15 min. The figure illustrates representative cells at different time points during the experiment. Scale bar = 10 μm.

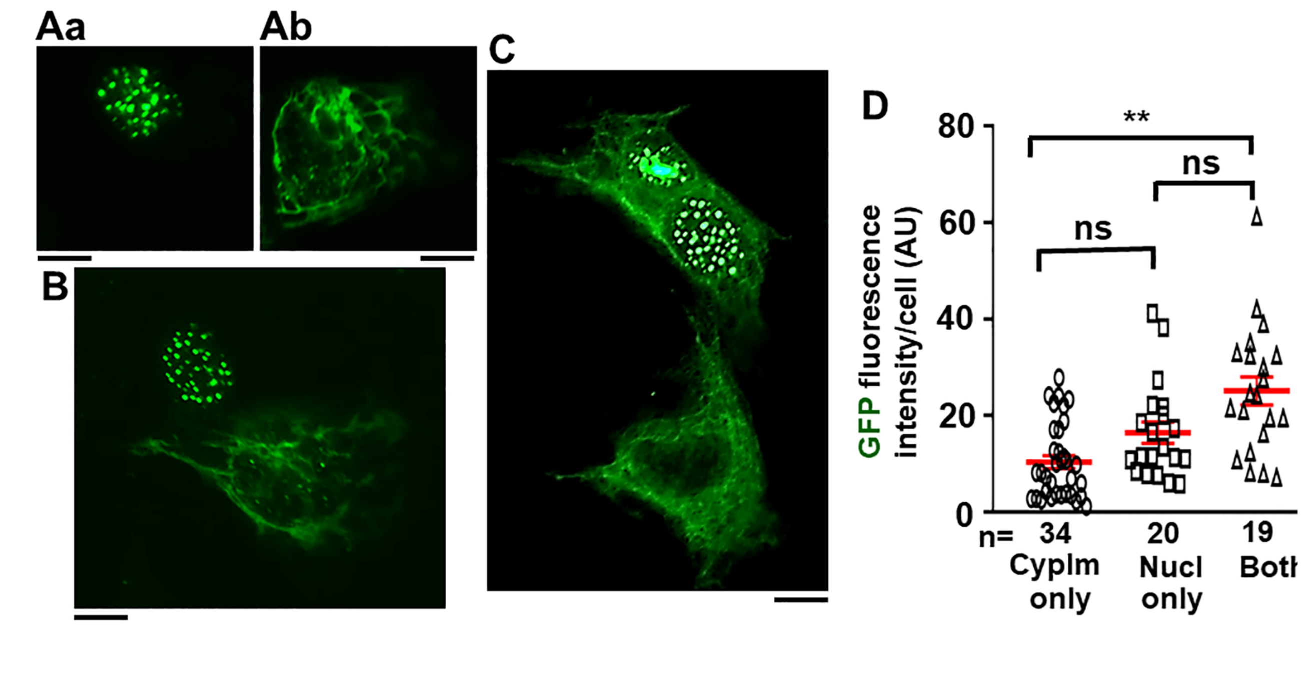

Fig. S2. Phenotypes of GFP-Mx1 structures in Huh7 cells. Huh7 cells were transiently transfected with the GFP-Mx1 expression vector. Two days later the cultures fixed and stained with DAPI. Subsequently multiple images were collected of GFP fluorescence using the same exposure setting (300 ms); DAPI staining as used to confirm cellular distribution of the GFP (nuclear or cytoplasmic or both). Panels A, B and C illustrate representative cellular phenotypes of GFP-Mx1 structures observed. Panels Aa and Ab are from the same image. All scale bar = 10 µm. In this experiment the distribution of cells with only nuclear, only cytoplasmic or both nuclear and cytoplasmic GFP-Mx1 was 58%, 27% and 15% respectively. Panel D: Summary of quantitation of GFP fluorescence intensity/cell (in arbitrary units, AU). Images were collected at the same exposure setting (300 ms) for all cells and quantitated using Image J (red lines indicate mean ± SE). n = number of cells evaluated per group, ns, not significant by ANOVA, **, $P < 0.001$.

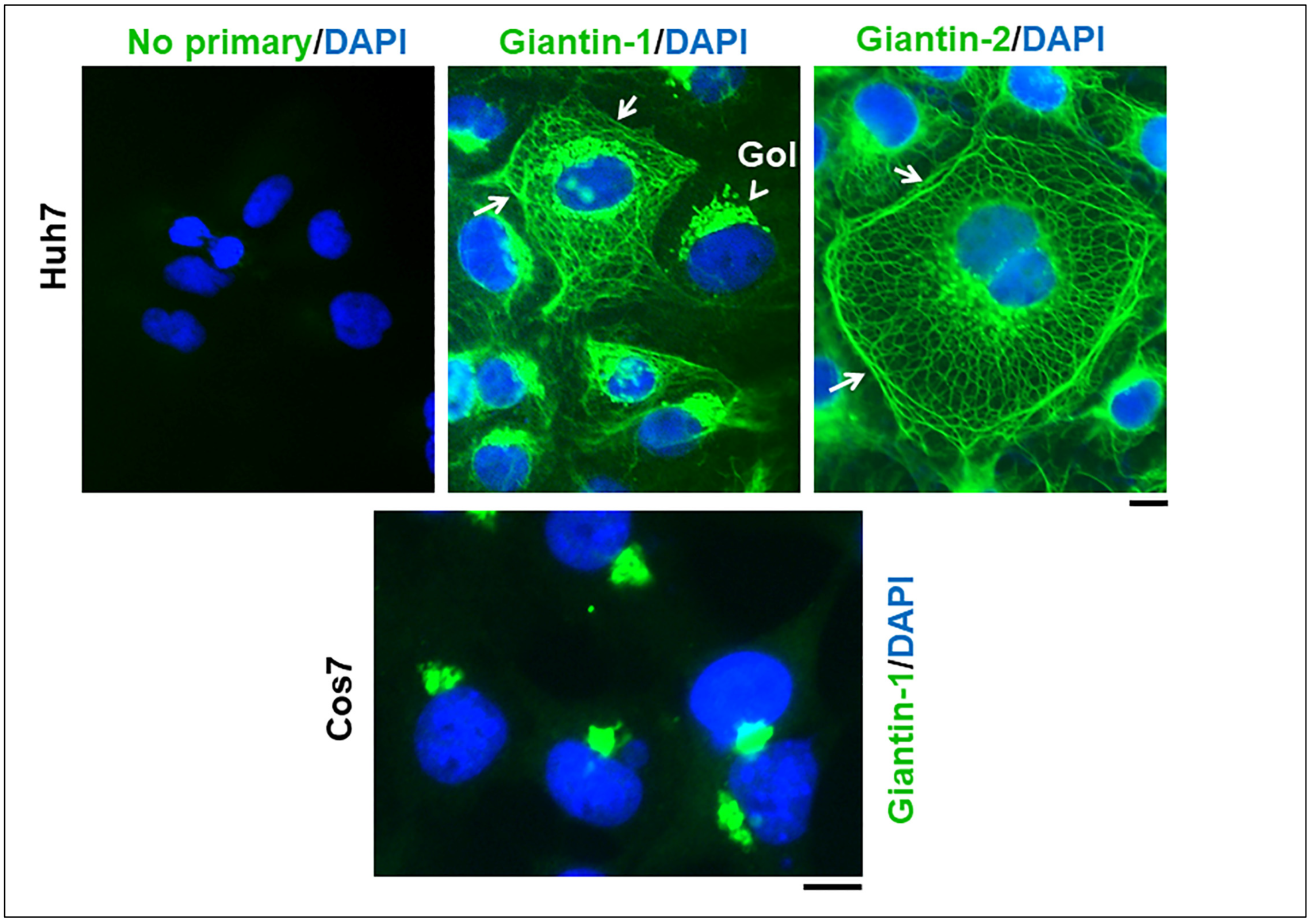

Fig. S3. Giantin-based cytoplasmic filamentous meshwork in Huh7 hepatocytes. Huh7 and Cos7 cells grown in 35 mm culture dishes for 2 days were fixed using 4% paraformaldehyde, permeabilized using the 0.05% saponin/ 0.05% digitonin buffer (33) and immunostained with two different anti-giantin rabbit pAbs (giantin-1 was from Abcam, giantin-2 was from Biolegend) or processed without a primary antibody. All cultures were then exposed to AlexaFluor 488-tagged donkey anti-rabbit pAb and then DAPI staining. The cultures were imaged using three-color fluorescence. "Gol" points to the Golgi apparatus, white arrows point out the cytoplasmic filamentous meshwork in Huh7 cells. Scale bar = 10 µm.

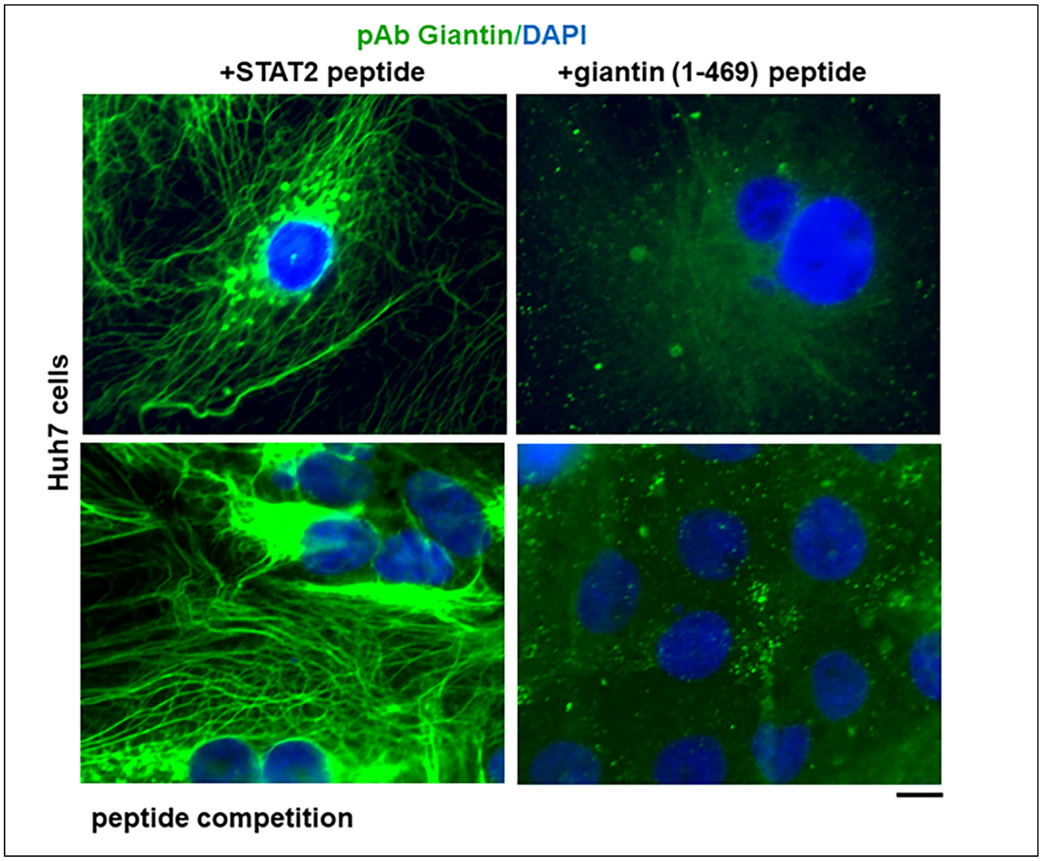

Fig. S4. Peptide competition assay showing specificity of the giantin-based immunostaining of the cytoplasmic filamentous meshwork. Huh7 cells grown in 35 mm plates for 2 days were fixed using 4% paraformaldehyde, and permeabilized using the 0.05% saponin/0.05% digitonin buffer (33). Aliquots of anti-giantin pAb (Biolegend) were incubated with either STAT2 peptide or the relevant giantin (1-468) peptide for 30 min on ice and then used to carryout immunostaining of the respective cultures as in Fig. S3. The respective cultures were imaged at identical microscope settings. Scale bar = 10 µm.

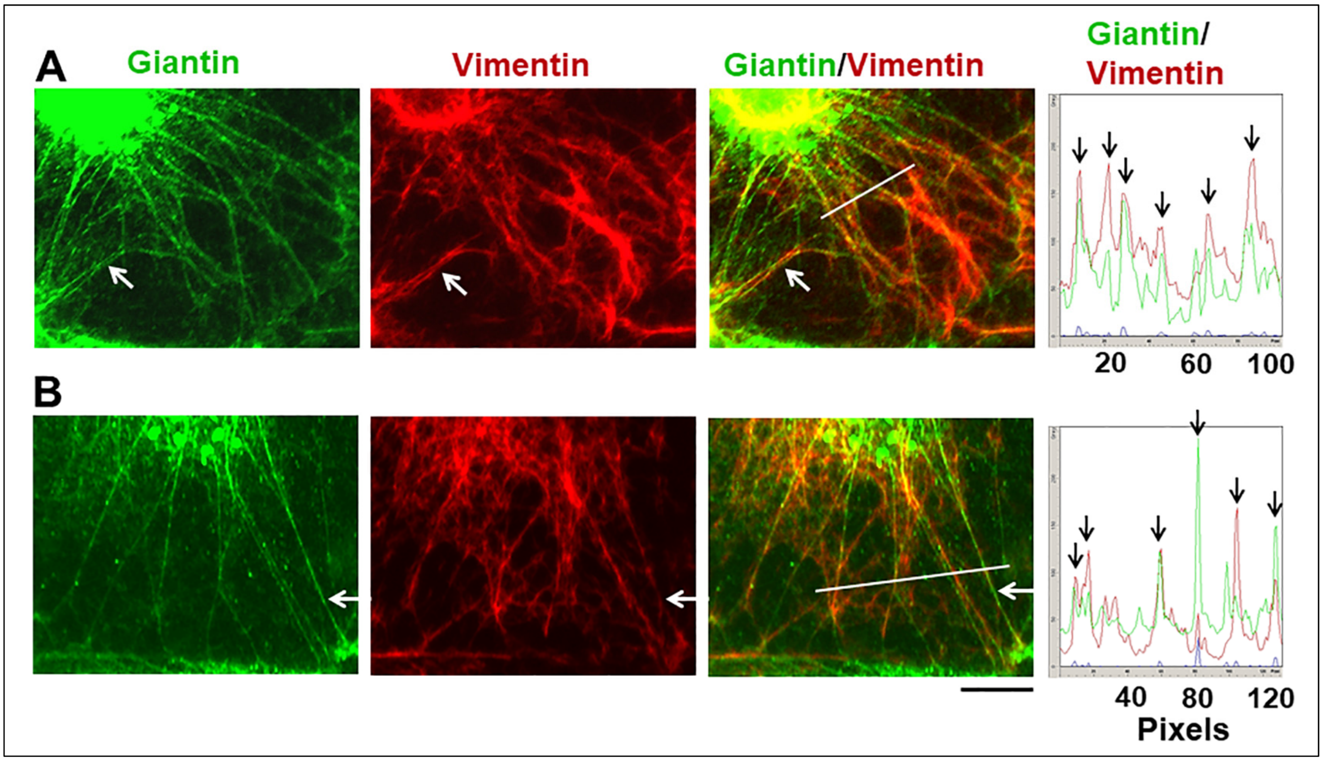

Fig. S5. Identification of giantin-filaments in Huh7 cells as intermediate filaments. Huh7 cells grown in 35 mm plates for 2 days were fixed using 4% paraformaldehyde, permeabilized using the 0.05% saponin/0.05% digitonin buffer, and then subjected to double immunofluorescence analysis for vimentin and giantin in a sequential manner (33). Panels A and B illustrate two different cells. White arrows placed within the images point to filaments that are giantin and vimentin double positive. The plots on the right enumerate fluorescence intensity of red and green pixels along the white line in the respective merged images; black arrows point to giantin and vimentin double positive filaments in the respective scans. Scale bar = 10 μm.

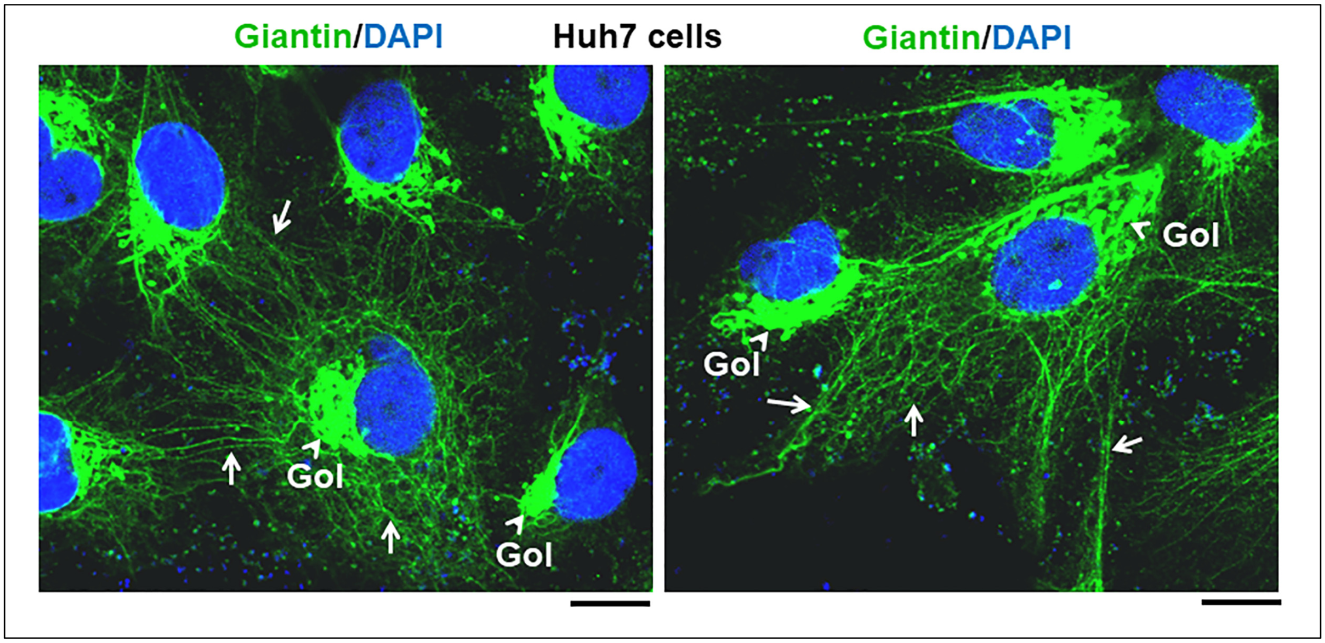

Fig. S6. Giantin filaments extend between adjacent Huh7 cells. Huh7 cells grown in 35 mm plates for 2 days were fixed using 4% paraformaldehyde, permeabilized using the 0.05% saponin/0.05% digitonin buffer, and then subjected to immunofluorescence analysis for giantin followed by imaging using a high-resolution confocal microscope (33). Panels A and B illustrate two different regions of the culture. White arrows point to giantin-positive filaments many of which extend between adjacent cells (left panel). "Gol" points to the Golgi apparatus. Scale bar = 10 µm.
